# Protective effects of Qing-Re-Huo-Xue formula on bleomycin-induced pulmonary fibrosis through the p53/IGFBP3 pathway

**DOI:** 10.1186/s13020-023-00730-y

**Published:** 2023-03-30

**Authors:** Fangyong Yang, Wenjing Du, Zhao Tang, Ying Wei, Jingcheng Dong

**Affiliations:** 1 Department of Integrative Medicine, Huashan Hospital, Fudan University, No. 12 Middle Wulumuqi Road, Jing’an District, Shanghai, 200040 China; 2 Institutes of Integrative Medicine, Fudan University, Shanghai, China

**Keywords:** Qing-Re-Huo-Xue formula, Pulmonary fibrosis, Inflammation, EMT, TMT proteomics, p53/IGFBP3 pathway

## Abstract

**Background:**

Idiopathic pulmonary fibrosis (IPF) is a chronic, progressive fibrosing lung disease with high mortality. Inflammation and epithelial mesenchymal transformation (EMT) may play an important role in the occurrence and development of IPF. Qing-Re-Huo-Xue formula (QRHXF) has been used clinically by our team for half a century and has obvious therapeutic effects on lung disease. Nevertheless, the role and mechanism of QRHXF in the treatment of IPF have never been studied.

**Methods:**

A mouse pulmonary fibrosis model was established by intratracheal injection of BLM. The effects of QRHXF on the treatment of pulmonary fibrosis were studied by pulmonary function testing, imaging examination, pathological staining, transmission electron microscopy (TEM) observation and mRNA expression. Tandem mass tag (TMT)-based quantitative proteomics was carried out to analyse the lung protein expression profiles between the control (CTL), bleomycin (BLM) and QRHXF (BLM + QRHXF) groups. Immunohistochemistry and qRT-PCR were used to verify the possible existence of drug target proteins and signalling pathways.

**Results:**

The results of pulmonary function, lung pathology and imaging examinations showed that QRHXF could significantly alleviate BLM-induced pulmonary fibrosis in vivo. Additionally, inflammatory cell infiltration and EMT were markedly reduced in BLM-induced PF mice administered QRHXF. Proteomics detected a total of 35 proteins, of which 17 were upregulated and 18 were downregulated. A total of 19 differentially expressed proteins (DEPs) overlapped between the BLM versus CTL groups and the BLM + QRHXF versus BLM groups. The expression of p53 and IGFBP3 was reversed in the QRHXF intervention group, which was verified by immunohistochemistry and qRT-PCR.

**Conclusions:**

QRHXF attenuated BLM-induced pulmonary fibrosis, and regulation of the p53/IGFBP3 pathway might be associated with its efficacy, which holds promise as a novel treatment strategy for pulmonary fibrosis patients.

**Graphical Abstract:**

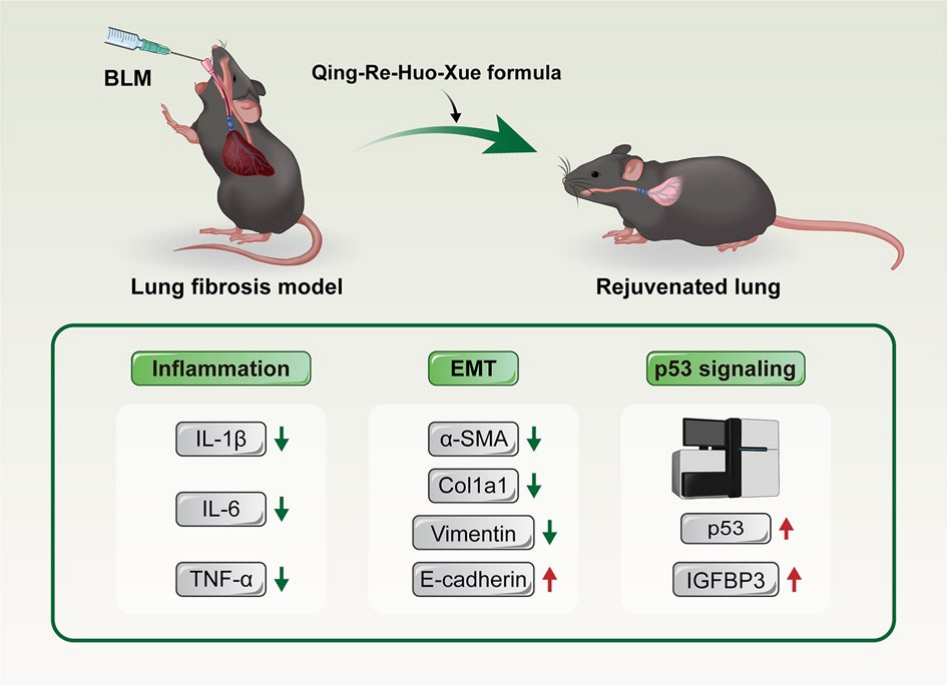

**Supplementary Information:**

The online version contains supplementary material available at 10.1186/s13020-023-00730-y.

## Background

Interstitial lung diseases (ILDs) are a challenging group of diffuse pulmonary parenchymal diseases with relatively high morbidity and mortality that are characterized by different extents of fibrotic and/or inflammatory abnormalities in the alveolar space or lung interstitium [1]. Idiopathic pulmonary fibrosis (IPF) accounts for 20–50% of ILDs and is the most serious type of interstitial lung disease (ILD) in idiopathic interstitial pneumonia (IIP). The median survival time after diagnosis with IPF is 2–4 years [2, 3]. Throughout most regions of the world, the incidence of IPF varies within the range of 0.22–93.7 per 100,000 people per year [4]. However, the incidence seems to be increasing in most countries [4]. Although much research has been performed, the pathogenesis of IPF is complex and incompletely understood and is known to be mainly related to inflammatory cell recruitment, fibroblast proliferation, and extracellular matrix (ECM) expansion [5]. Currently, treatment options for pulmonary fibrosis (PF) are very limited, and lung transplantation is the only effective approach for patients with advanced PF and respiratory failure [6]. Pirfenidone (PFD) and nintedanib are the only two anti-PF drugs currently approved by the United States Food and Drug Administration (FDA). Although they are effective in slowing the decline in pulmonary function, they have no significant effect on reducing mortality, improving respiratory symptoms, or improving patients’ quality of life [7–9]. Thus, exploring effective and safe therapies for pulmonary fibrosis has become particularly urgent.

Qing-Re-Huo-Xue formula (QRHXF), which is composed of *Scutellaria baicalensis* (the root of *Scutellaria baicalensis* Georgi, described as Huang-Qin in Chinese herbal medicine) and *Radix Paeonia Rubra* (the dried root of *Paeoniae lactiflora* Pall, Sichuan, China, described as Chi-Shao in Chinese herbal medicine), is an empirical traditional Chinese herbal compound prescription by Prof. Jingcheng Dong. Basic and clinical research has been conducted with QRHXF for nearly half a century, and it has been widely used to treat respiratory diseases in the clinic by our team. Our previous research found that QRHXF and its active components have obvious therapeutic effects on lung diseases such as asthma, chronic obstructive pulmonary disease (COPD), and lung cancer [10–13]. Furthermore, some studies have demonstrated that the active components of QRHXF have the ability to inhibit PF [14–16]. Nevertheless, the role and mechanism of QRHXF in the treatment of pulmonary fibrosis have never been investigated.

Traditional Chinese medicine (TCM) has the characteristics of multiple components, pathways, and targets, so research on its potential mechanism of action is an arduous task. Proteomics, as a targeted molecular technology, plays a substantial role in the development of therapeutic drugs and the exploration of involved potential molecular mechanisms [17]. The integrative, dynamic, and networked characteristics of proteomics are consistent with the holistic and dialectical concepts of TCM. Therefore, proteomics has received increasing attention and has been widely used in the field of TCM. With the advantages of high flux, high resolution, good repeatability, accurate quantification, rich data, and a high degree of automation, liquid chromatography-tandem mass spectrometry (LC‒MS/MS)-based tandem mass tag (TMT) labelling quantitative proteomics is increasingly applied in various studies [18, 19].

In this study, we assessed the curative efficiency of QRHXF on bleomycin (BLM)-induced PF mice and further explored the potential mechanism using TMT-LC‒MS/MS. Analysing the efficacy of QRHXF by quantitative proteomic technology might provide new insight into the treatment of PF.

## Methods and materials

### Preparation of QRHXF and chemical constituent identification

Qing-Re-Huo-Xue formula (QRHXF), which is composed of *Scutellaria baicalensis* (batch number: 20210801) and *Radix Paeoniae Rubra* (batch number: 20210501), was purchased from Hejitang Pharmaceutical Group Co., Ltd. (Anhui, China). The clinical dosage of QRHXF was 50 g of raw herbs/kg body weight of humans, and the ratio of the two herbs was 3:2 (w/w). Thus, the final concentrations of gavage administration were equivalent to 7.5 g of raw herbs/kg body weight of mice according to the dose conversion between mice and humans [20]. QRHXF was immersed in water for 30 min and decocted for 30 min. Then, the decoction was filtrated and concentrated to the extract with a rotary evaporator and stored at − 20 °C for 24 h. After vacuum freeze-drying, it was preserved at 4 °C and dissolved in distilled water before use. High-performance liquid chromatography quadrupole time-off flight mass spectrometry ultraviolet (HPLC-Q/TOF–MS-UV) was used to separate and identify the chemical ingredients of QRHXF by our research team, as described in Additional file 1: Fig. S1 and Additional file 2: Fig. S2.

### Reagents and antibodies

BLM was supplied by Hisun Pharmaceutical Co., Ltd. (Zhejiang, China). PFD was obtained from MedChemExpress LLC (NJ, USA). Enzyme-linked immunosorbent assay (ELISA) kits for IL-1β (EM3184M), IL-6 (EM3201M), TNF-α (EM3311M), and TGF-β1 (EM3285M) were provided by Shanghai WellBio Technology Co., Ltd. (Shanghai, China). Primary antibodies against α-SMA (19245) and vimentin (5741) were purchased from Cell Signal Technology Inc. (MA, USA). Antibodies against p53 (A3185) and IGFBP3 (A16052) were acquired from ABclonal Technology Co., Ltd. (Wuhan, China).

### Animal model and drug treatment

**Fig. 1:**
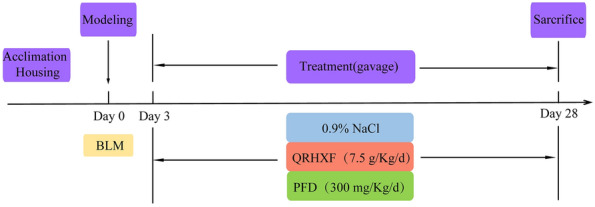
Flow chart of PF mouse modelling and drug intervention. Beginning on the 3rd day after BLM treatment, QRHXF (7.5 g raw herbs/kg) or PFD (300 mg/kg) was administered by gavage once daily for 25 days. Mice in the control and model groups were administered 0.9% saline daily via gastric gavage

C57BL/6 mice (male, 8 weeks old) were supplied by Beijing Vital River Laboratory Animal Technology Co., Ltd. (Beijing, China) and kept at Fudan University under specific pathogen-free (SPF) conditions. Mice were kept at a temperature of 25 ± 2 °C under a light/dark cycle of 12 h and were given ad libitum access to water and food. All procedures were strictly carried out according to the ethical guidelines of the Fudan University Animal Care and Use Committee. After 7 days of fostering, 32 mice were randomly assigned to four groups (8 mice/group): the control group (CTL), model group (BLM), QRHXF treatment group (BLM + QRHXF), and pirfenidone treatment group (BLM + PFD). After anaesthesia with pentobarbital sodium (50 mg/kg), the PF mouse model was constructed by intratracheal administration of 3.5 U/kg BLM using an intratracheal quantitative drug delivery device (Yuyan Instruments Co., Ltd. Shanghai China). Beginning on the 3rd day after BLM treatment, QRHXF (7.5 g raw herbs/kg) or PFD (300 mg/kg) was administered by gavage once daily for 25 days. Mice in the control and model groups were administered 0.9% saline daily via gastric gavage. The process of PF mouse modelling and drug intervention is shown in Fig. 1.

### Small-animal PET/CT scanning

Four mice in each group were randomly selected for successive small-animal PET/CT with (18)F-FDG at D27. Mice were intravenously injected (tail vein) with 5 MBq of (18)F-FDG 20 min before imaging. The mice were then maintained under anaesthesia (1.5%) and placed on the imaging heating bed for PET/CT (Inveon; Siemens Co., USA). PET scanning began 60 min after the tracer injection and lasted 30 min. At the end of the PET acquisition (30 min), a CT scan of the same area (the central lung area) was performed. We calculated the SUV_mean_ for the lungs of each mouse. The mean standardized uptake value (SUV_mean_) was calculated in MBq/mL (mean activity within each region of interest) by the ratio A0 [injected activity decay-corrected at the start of the PET acquisition (MBq) to animal weight (g)].

### Pulmonary function analysis

Twenty-four hours after the PET/CT inspection, the mice were anaesthetized with an intraperitoneal injection of 2% pentobarbital sodium (80 mg/kg) and underwent a tracheotomy. Next, the pulmonary function test (PFT) was conducted using the PFT system (DSI, Buxco, USA). Forced vital capacity (FVC), chord compliance (Cchord), inspiratory capacity (IC), and peak expiratory flow (PEF) were acquired to evaluate pulmonary function in mice.

### Cell counting of bronchoalveolar lavage fluid (BALF) and ELISA

BALF was acquired by lavage of the lung with cold PBS (three aliquots of 300 μL) and centrifuged (2000 rpm, 15 min, 4 °C) to obtain the supernatant. PBS was added to the lower cells of BALF for resuspension, and the cells were classified and calculated using an automated cell counter (Mindray, China). Mouse blood was obtained from the eyeballs, set aside for 2 h at room temperature, and then centrifuged (4000 rpm, 30 min, 4 °C) to obtain the supernatant. Both BALF and serum were collected and preserved at − 80 °C for further ELISAs. Pro-inflammatory cytokines, including IL-1β, IL-6, and TNF-α, and the pro-fibrotic factor TGF-β1 in BALF and serum were quantified by ELISA kits (WellBio Technology Co., Ltd, Shanghai, China) under the guidance of the manufacturer’s instructions.

### Histopathology and immunohistochemistry

The left middle lungs were fixed with 10% formalin buffer, dehydrated with an alcohol gradient, and embedded in paraffin. After deparaffinization and rehydration, lung sections (5 µm thickness) were stained with haematoxylin and eosin (H&E) solution, Masson’s trichrome solution, and Sirius Red solution. Quantitative histologic analysis of fibrosis was performed by ImageJ.

In brief, after removal of the paraffin and dehydration of paraffin-embedded lung slides, sections of lungs (5 μm) were soaked in 3% H_2_O_2_ for 25 min at 37 °C to remove endogenous peroxidase. Afterwards, the sections were sealed with 10% goat serum for 30 min at 37 °C and then incubated with diluted primary antibodies overnight at 4 °C. After washing by shaking on the decolorizing shaker 3 times with PBS, slides were incubated with a suitable secondary antibody (goat anti-rabbit IgG) for 50 min at room temperature. Then, the sections were developed with DAB colour developing solution and rinsed with tap water to stop the reaction. Finally, the sections were mounted with coverslips after counterstaining with haematoxylin staining solution and examined under a light microscope.

### Transmission electron microscopy (TEM)

Lung tissue blocks (1 mm^3^) were rinsed with PBS and fixed with 1% O_S_O_4_ for 2 h at room temperature. Then, the blocks were dehydrated with gradient alcohol. Subsequently, the tissue blocks were embedded with 1:1 acetone:EMBed 812 (90529-77-4, SPI-Chem, PA, USA) for 2–4 h at 37 °C and 1:2 acetone:EMBed 812 overnight at room temperature. The resin blocks were cut into 60–80-nm ultrathin sections and then stained with 2% uranium acetate and 2.6% lead citrate. Finally, lung sections were observed and captured for further analysis by using TEM (Hitachi-7800, Tokyo, Japan).

### Tandem mass tag proteomics analysis

Twelve frozen lung tissues were thoroughly ground with liquid nitrogen and lysed by the addition of lysis buffer containing a protease inhibitor cocktail. Then, samples were further lysed with sonication (1 s/1 s intervals, 3 min time, and 80 W power) and centrifuged (15,000 × g, 15 min, 4 °C) to remove insoluble particles. Protein concentration determination experiments were performed according to the instructions of the BCA kit (Thermo Scientific, USA). Tandem mass tag proteomics analysis was conducted as previously described by our team [21]. All of the raw data were processed by ProteomeDiscoverer (v.2.4) and searched against the same database. The UniProt, GO, KEGG, and KOG/COG databases were used to extract the annotation information and explore the protein function. Unique peptides ≥ 1, fold change (FC) > 1.2, and *p* value < 0.05 were set as conditions for differentially expressed protein (DEP) screening.

### RNA extraction and real-time PCR analysis

Before the quantitative real-time PCR (qRT-PCR) experiment, whole RNA was extracted from lung tissues using TRIzol reagent (Invitrogen, USA). Then, reverse transcription was conducted to acquire cDNA using a reverse transcription kit (TAKARA, JPN). The cDNAs were amplified using the Genious 2X SYBR Green Fast qPCR Mix kit (ABclonal Technology Co., Ltd, Wuhan, China) and carried out on a real-time PCR instrument (QuantStudio 6 Flex, Thermo Fisher, USA). The primers used are listed in Additional file 4: Table S1.

### Statistical analysis

The data are presented as the mean ± standard deviation (SD). Differences among groups were investigated by one-way analysis of variance (ANOVA) followed by Tukey’s post hoc test with GraphPad Prism 8.0 (GraphPad Software Inc., CA, USA). The differences were considered statistically significant when the *p* value < 0.05.

## Results

### QRHXF-treated mice are protected against BLM-induced PF

**Fig. 2:**
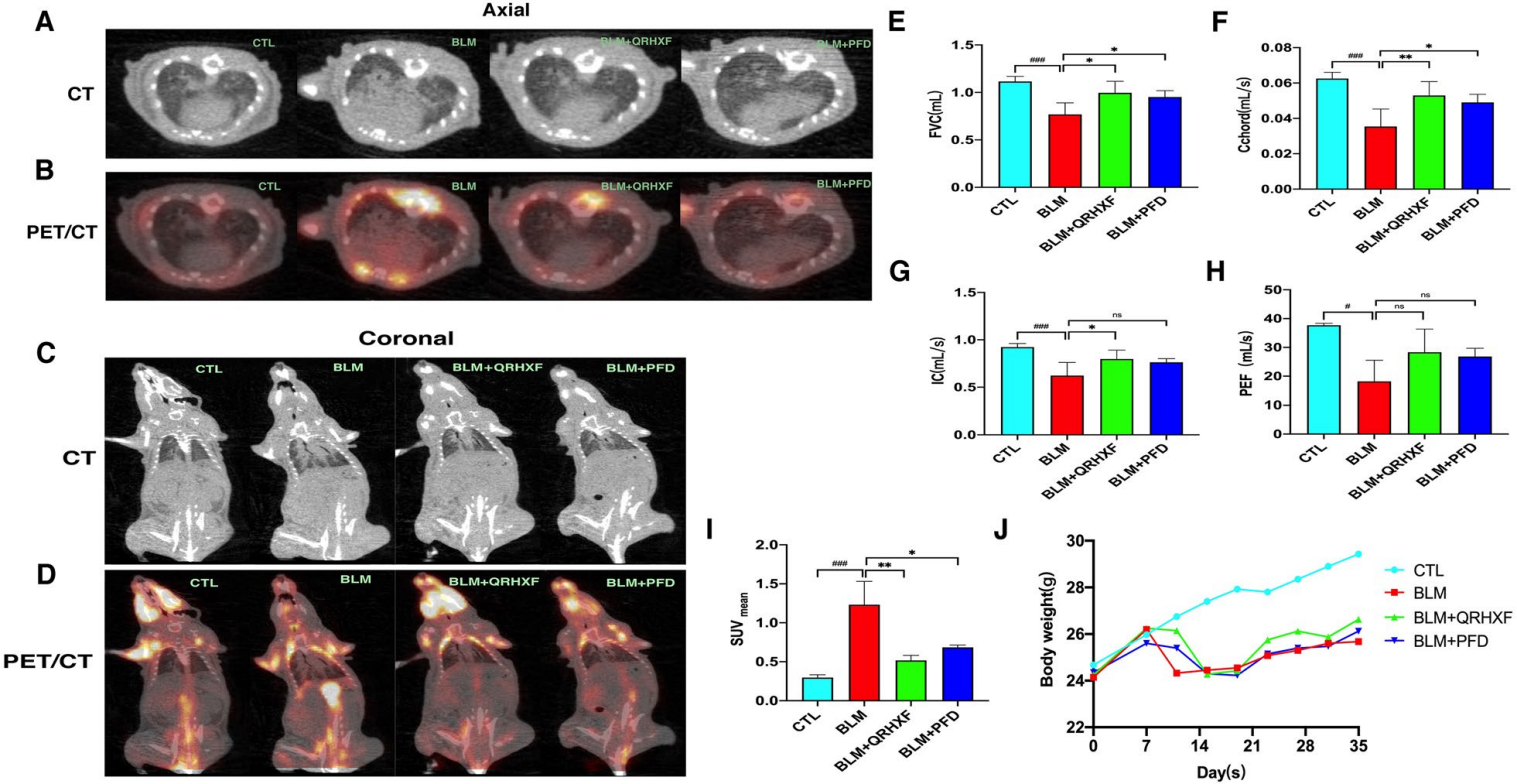
QRHXF-treated mice are protected against BLM-induced PF. **A** CT of axial sections. **B** PET/CT of axial sections.** C** CT of coronal sections.** D** PET/CT of coronal sections.** E**–**H** PFTs (FVC, Cchord, IC and PEF) were measured.** I** Lung SUV_mean_ of the CTL, BLM, BLM + QRHXF, and BLM + PFD groups. n = 4 mice/group, ^#^*p* < 0.05, ^###^*p* < 0.001, compared with the CTL group; *p < 0.05, **p < 0.01, compared with the BLM group.** J** Body weights were recorded every 3 days until the mice were sacrificed. n = 6 mice/group. All data are presented as the mean ± SD

Pulmonary CT of BLM-treated mice at D27 showed a wide range of patchy, fibrous cord and even consolidated changes in comparison with the control group. Interestingly, the patchy and fibrous cord shadows were significantly reduced, and the degree of fibrosis decreased in the QRHXF group (Fig. 2a, c). From the (18)F-FDG PET/CT imaging findings (Fig. 2b, d), we found that the uptake of (18)F-FDG in the lung tissue of the BLM group was significantly higher, and QRHXF treatments decreased this uptake. Furthermore, the quantitative results showed that the average SUV value of BLM group mice increased significantly, and treatment with QRHXF reduced the average value (Fig. 2i). A pulmonary function test was performed on D28. The results showed that mice in the BLM group showed a decrease in FVC, Cchord, IC, and PEF compared with those of the control group. Treatment with QRHXF induced an obvious increase in the above three indicators, but the improvement in PEF was not obvious (Fig. 2e–h). Measurement of the body weights was conducted every 4 days until the mice were sacrificed. As presented in Fig. 2j, BLM intervention resulted in significant weight loss in mice, which was ameliorated by 25 days of QRHXF or PFD treatment.

### Effects of QRHXF on the histopathology in BLM-induced PF

**Fig. 3:**
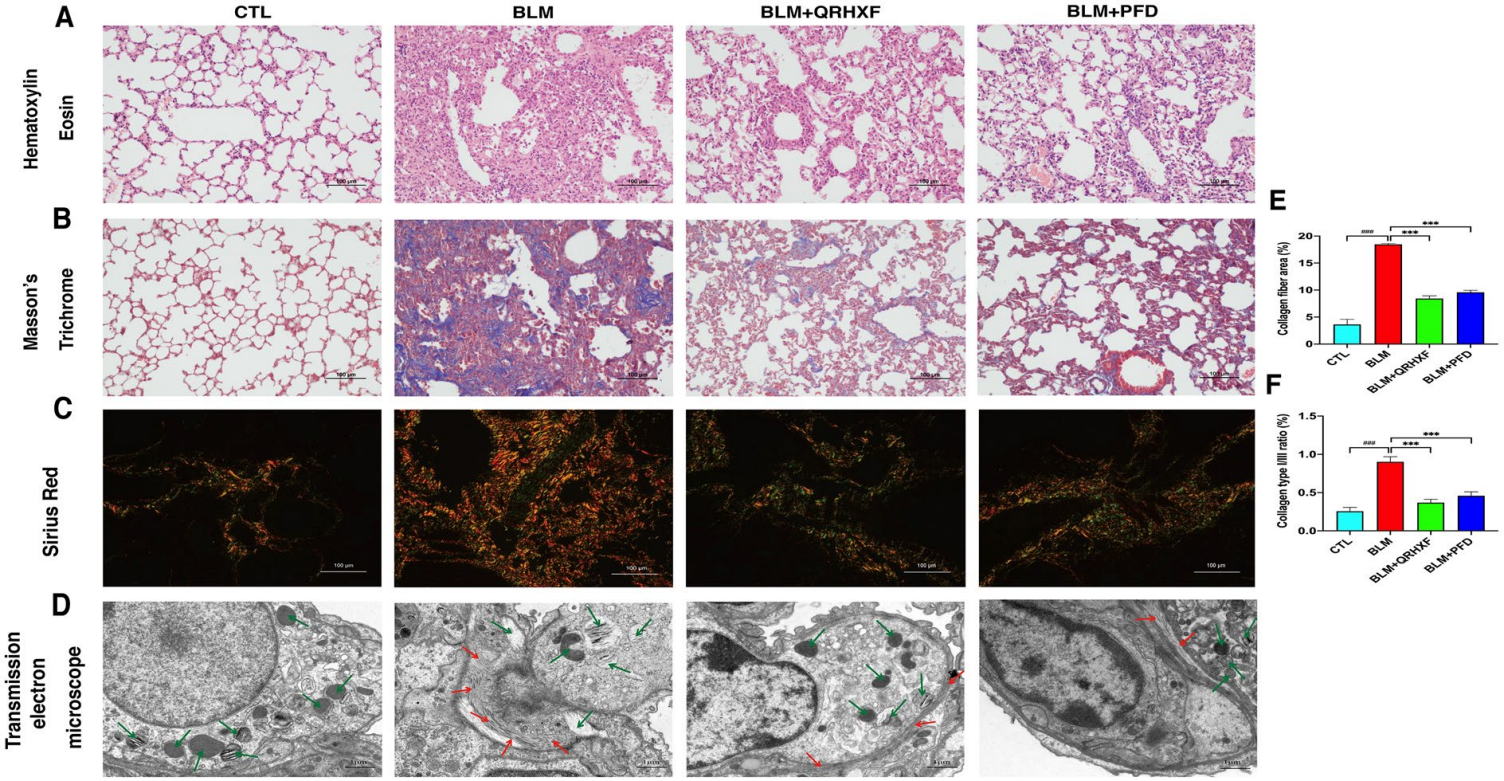
Effects of QRHXF on the histopathology in BLM-induced PF. Representative images of treated mouse lungs stained with **A** H&E, **B** Masson’s trichrome, and **C** Sirius Red. **D** Transmission electron microscope, lamellar bodies and multivesicular bodies (green arrow), and collagen deposition (red arrow). ImageJ software was used to quantify **E** Masson’s trichrome staining of the lung sections from the different groups and **F** the ratio of type I/III collagen of Sirius Red staining of the lung sections from the different groups. n = 4 mice/group, ^###^*p* < 0.001, compared with the CTL group, ***p < 0.001, compared with the BLM group. All data are presented as the mean ± SD

Compared to that of the control group, H&E staining of the pulmonary tissue in the BLM-treated group displayed obvious morphological changes and severe damage, including destruction of normal pulmonary architecture, dramatic thickening of the alveolar septum, and enormous filling of fibrous tissue. However, after treatment with QRHXF, pulmonary patchy and fibrous cord shadows were significantly reduced, and the degree of fibrosis was significantly reduced compared with BLM-treated model mice (Fig. 3a). Consistent with these results, the results of Masson’s trichrome and Sirius Red staining showed that QRHXF treatment significantly reduced collagen deposition in BLM-treated mouse lung tissue (Fig. 3b, c). The ratio of collagen area and collagen type I/III in Masson’s trichrome- and Sirius Red-stained lung sections also confirmed this finding (Fig. 3e, f). Alveolar epithelial type II cells (AEC 2) are essential for maintaining alveolar integrity and function. TEM was used to observe the ultrastructural changes in AEC 2 after QRHXF treatment. AEC 2 in the lung tissues of the control mice had an intact structure, with complete lamellar bodies and multivesicular bodies (green arrow). The structure of AEC 2 in BLM-treated mouse lung tissue was destroyed, including swelling and vacuolation of lamellar bodies and abundant collagen deposition in the stroma (red arrow). QRHXF treatment significantly improved the ultrastructural changes induced by BLM (Fig. 3d).

### QRHXF suppresses BLM-induced pulmonary inflammation

**Fig. 4:**
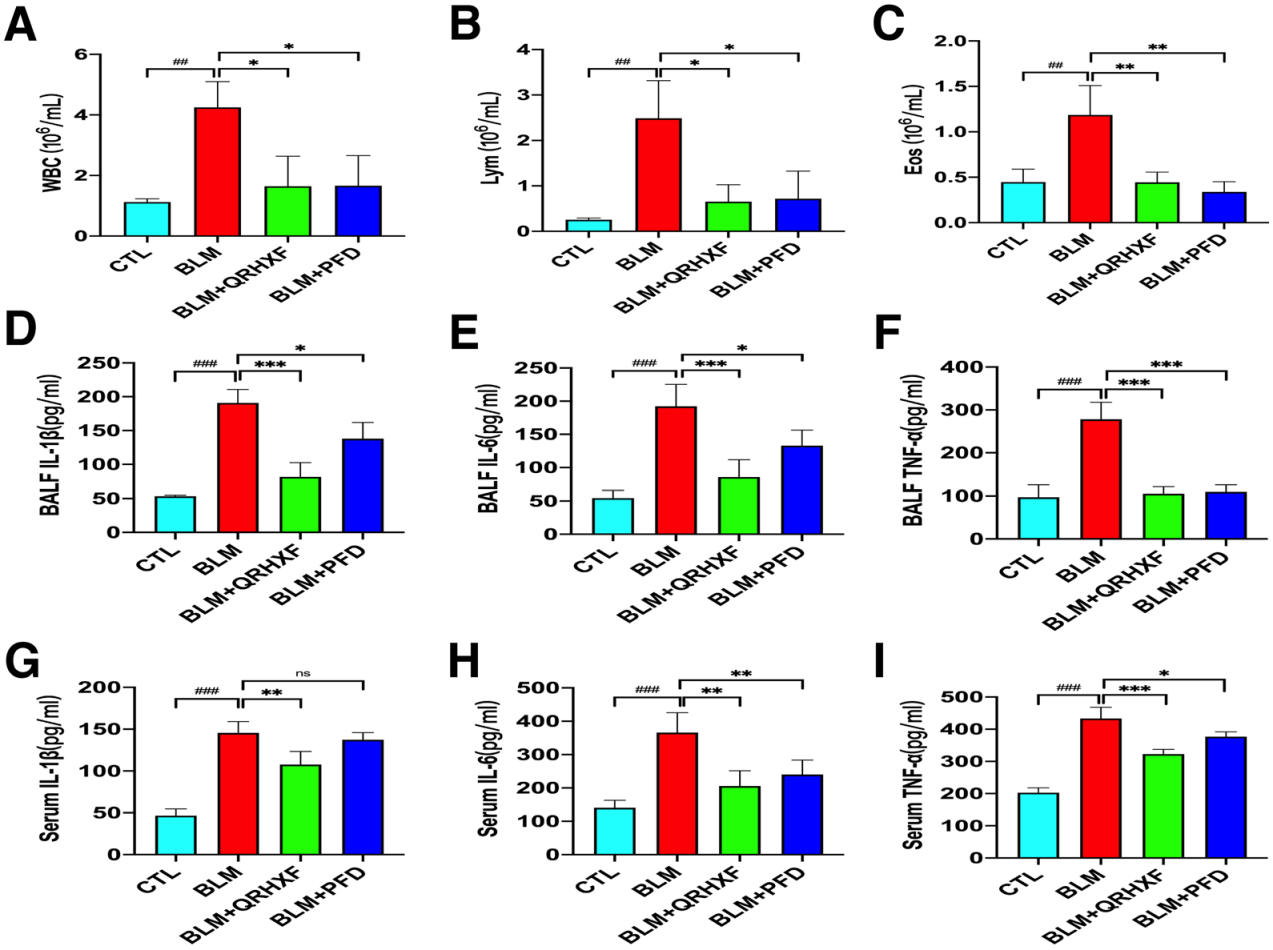
QRHXF suppresses BLM-induced pulmonary inflammation. **A–C** Inflammatory cells (neutrophils, lymphocytes, and eosinophils) in BALF. Inflammatory cytokines (IL-1β, IL-6, and TNF-α) were quantified by ELISA in **D–F** BALF and in **G–I** serum. n = 4 mice/group, ^##^*p* < 0.001, ^###^*p* < 0.001, compared with the CTL group, *p < 0.05, **p < 0.01, ***p < 0.001, compared with the BLM group. All data are presented as the mean ± SD

The anti-inflammatory effects of QRHXF were evaluated by inflammatory cell counting and ELISA. As depicted in Fig. 4a–c, mice treated with BLM displayed notable infiltration of inflammatory cells in the lungs, such as neutrophils, lymphocytes, and eosinophils (*p* < 0.005). QRHXF significantly reduced the cell populations. Additionally, inflammatory cytokines, including IL-1β, IL-6, and TNF-α, in BALF increased dramatically in BLM-treated mice compared with normal mice and were significantly reduced by treatment with QRHXF or PFD (Fig. 4d–f). Consistent with the BALF results, inflammatory cytokines, including IL-1β, IL-6, and TNF-α, in serum were also significantly reduced by QRHXF or PFD treatment (Fig. 4g–i). Thus, these outcomes indicated that QRHXF could attenuate the lung inflammation induced by BLM.

### QRHXF inhibits BLM-induced EMT in mice

**Fig. 5:**
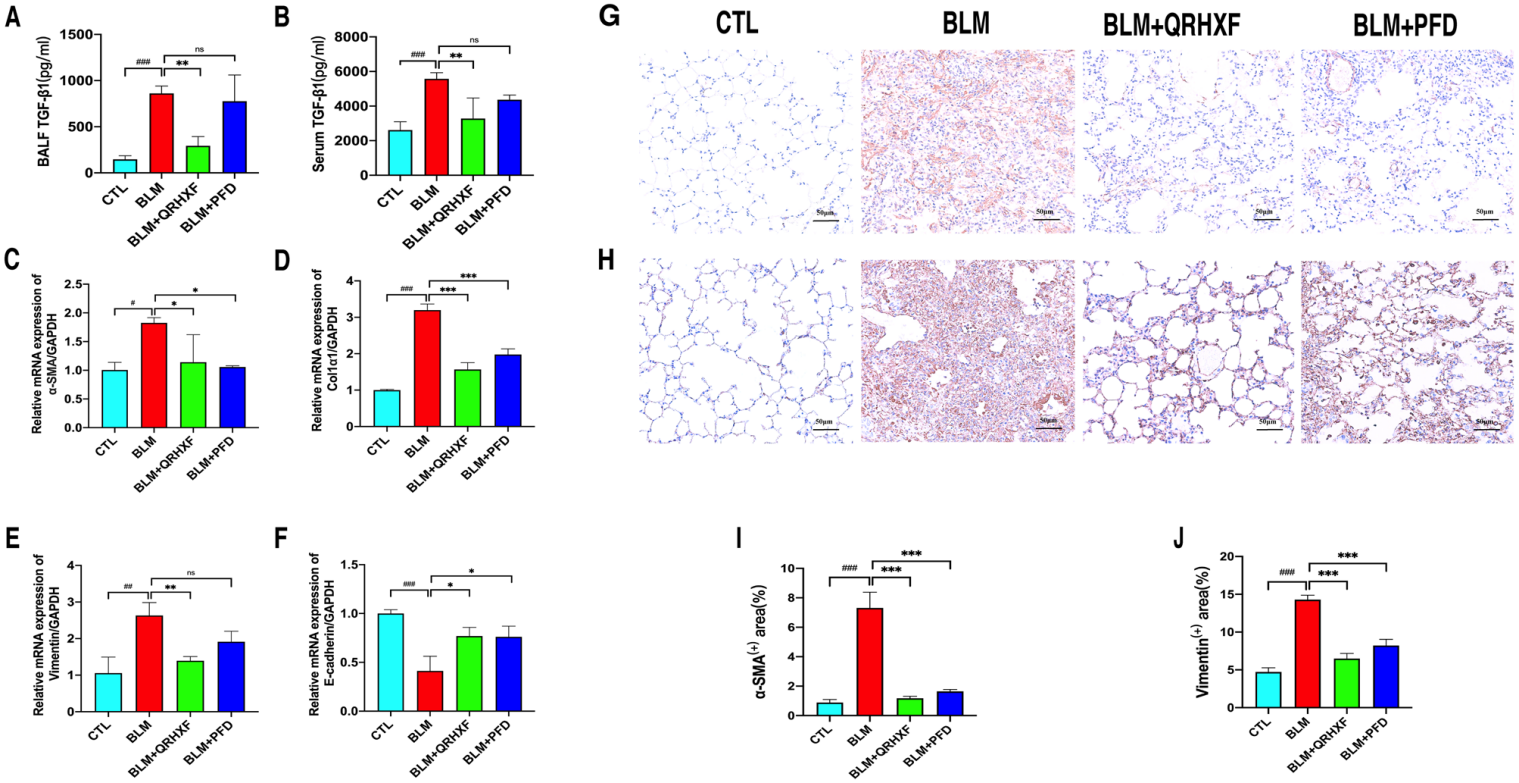
QRHXF inhibits BLM-induced EMT in mice. **A** TGF-β1 level in BALF. **B** TGF-β1 level in serum. qRT-PCR analysis of the relative mRNA expression of EMT factors, including** C** α-SMA,** D** Col1a1,** E** vimentin, and** F** E-cadherin. Immunohistochemistry staining detection for the protein expression of EMT factors, including** G** α-SMA staining in lung tissue and **H** vimentin staining in lung tissue. Quantitative analysis of** I** α-SMA and **J** vimentin. n = 4 mice/group, ^#^*p* < 0.05, ^##^*p* < 0.001, ^###^*p* < 0.001, compared with the CTL group, *p < 0.05, **p < 0.01, ***p < 0.001, compared with the BLM group. All data are presented as the mean ± SD

EMT is one of the important mechanisms of fibrosis and plays a vital role in the development of PF. TGF-β1 is not only a key factor involved in the occurrence of PF but is also an effective inducer of EMT. In comparison with expression in mice in the control group, the expression of TGF-β1 in both BALF and serum was significantly increased in mice following BLM intervention, and QRHXF or PFD treatment significantly reduced this level (Fig. 5a, b). Common indicators of EMT, such as *α-SMA*, *Col1a1*, *vimentin* and *E-Cadherin*, were analysed by qRT-PCR analysis. As shown in Fig. 5c–f, the relative mRNA expression levels of *α-SMA*, *Col1a1*, and *vimentin* increased, while the expression level of *E-cadherin* decreased dramatically after treatment with BLM, and QRHXF or PFD treatment changed these tendencies. Furthermore, immunohistochemistry staining of pulmonary tissue indicated that the expression of α-SMA and vimentin significantly increased in BLM-treated mice, and QRHXF treatment reduced these changes (Fig. 5g–j).

### Quantitative proteomics analyses to reveal PF proteins

**Fig. 6:**
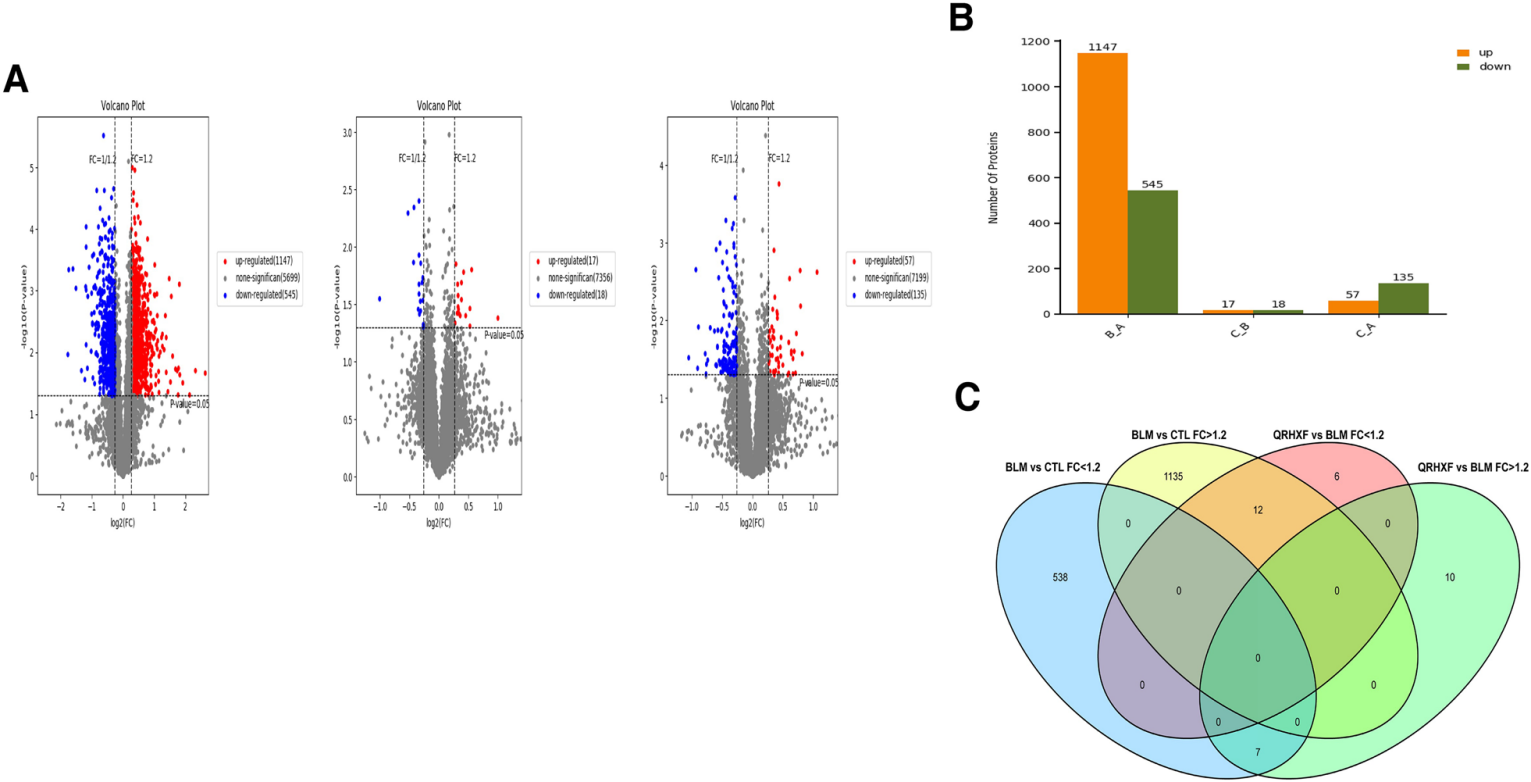
Identification of DEPs in lungs between the CTL, BLM and BLM + QRHXF groups. **A** Volcano plot of the BLM versus CTL group, BLM + QRHXF versus BLM group, and BLM + QRHXF versus CTL group. **B** Histogram of DEPs in the different comparison groups. **C** Venn diagrams showing the distribution of overlapping proteins between the BLM and CTL groups and the BLM and QRHXF + BLM groups

To further investigate the mechanism by which QRHXF inhibits PF, TMT quantitative proteomics and bioinformatics analysis were used to detect proteins in lung tissues in the CTL, BLM, and BLM + QRHXF groups (n = 4). FC > 1.2 and *p* value < 0.05 were set as the screening conditions of DEPs. A total of 1147 upregulated proteins and 545 downregulated proteins were identified in the BLM versus CTL groups. Moreover, 17 upregulated proteins and 18 downregulated proteins were found in the BLM + QRHXF versus BLM groups (Fig. 6a, b, Additional file 3: Fig. S3 and Additional file 5: Table S2). A total of 19 DEPs overlapped between the BLM and CTL groups and the BLM + QRHXF and BLM groups (Fig. 6c and Table 1).

**Table 1:** 19 DEPs regulated by treatment of QRHXF

Protein accession	Gene name	Protein description	QRHXF/BLM ratio	Regulated type	BLM/CTL ratio	Regulated type
Q8BLY1	Smoc1	SPARC-related modular calcium-binding protein 1 OS = Mus musculus OX = 10,090 GN = Smoc1 PE = 2 SV = 2	1.996	Up	0.299	Down
P47878	Igfbp3	Insulin-like growth factor-binding protein 3 OS = Mus musculus OX = 10,090 GN = Igfbp3 PE = 2 SV = 2	1.367	Up	0.574	Down
G3XA59	Lrrc32	Transforming growth factor beta activator LRRC32 OS = Mus musculus OX = 10,090 GN = Lrrc32 PE = 1 SV = 1	1.259	Up	0.788	Down
Q8K177	Krtcap3	Keratinocyte-associated protein 3 OS = Mus musculus OX = 10,090 GN = Krtcap3 PE = 2 SV = 2	1.246	Up	0.755	Down
P70365	Ncoa1	Nuclear receptor coactivator 1 OS = Mus musculus OX = 10,090 GN = Ncoa1 PE = 1 SV = 2	1.301	Up	0.451	Down
Q99M04	Lias	Lipoyl synthase, mitochondrial OS = Mus musculus OX = 10,090 GN = Lias PE = 1 SV = 1	1.220	Up	0.807	Down
O55137	Acot1	Acyl-coenzyme A thioesterase 1 OS = Mus musculus OX = 10,090 GN = Acot1 PE = 1 SV = 1	1.236	Up	0.759	Down
Q99P65	Slc29a3	Equilibrative nucleoside transporter 3 OS = Mus musculus OX = 10,090 GN = Slc29a3 PE = 1 SV = 1	0.790	Down	1.251	Up
Q14CH0	Fam171b	Protein FAM171B OS = Mus musculus OX = 10,090 GN = Fam171b PE = 1 SV = 2	0.830	Down	1.328	Up
Q8K004	Spata2	Spermatogenesis-associated protein 2 OS = Mus musculus OX = 10,090 GN = Spata2 PE = 1 SV = 1	0.819	Down	1.453	Up
P02802	Mt1	Metallothionein-1 OS = Mus musculus OX = 10,090 GN = Mt1 PE = 1 SV = 1	0.496	Down	3.501	Up
O54791	Maff	Transcription factor MafF OS = Mus musculus OX = 10,090 GN = Maff PE = 2 SV = 1	0.693	Down	1.357	Up
A2ASZ8	Slc25a25	Calcium-binding mitochondrial carrier protein SCaMC-2 OS = Mus musculus OX = 10,090 GN = Slc25a25 PE = 1 SV = 1	0.739	Down	1.442	Up
O08997	Atox1	Copper transport protein ATOX1 OS = Mus musculus OX = 10,090 GN = Atox1 PE = 1 SV = 1	0.743	Down	1.438	Up
Q6P6L0	Filip1l	Filamin A-interacting protein 1-like OS = Mus musculus OX = 10,090 GN = Filip1l PE = 1 SV = 2	0.802	Down	1.395	Up
Q62159	Rhoc	Rho-related GTP-binding protein RhoC OS = Mus musculus OX = 10,090 GN = Rhoc PE = 1 SV = 2	0.808	Down	1.284	Up
Q9D819	Ppa1	Inorganic pyrophosphatase OS = Mus musculus OX = 10,090 GN = Ppa1 PE = 1 SV = 1	0.829	Down	1.424	Up
Q64449	Mrc2	C-type mannose receptor 2 OS = Mus musculus OX = 10,090 GN = Mrc2 PE = 1 SV = 3	0.791	Down	1.433	Up
Q80X90	Flnb	Filamin-B OS = Mus musculus OX = 10,090 GN = Flnb PE = 1 SV = 3	0.827	Down	1.339	Up

**Fig. 7:**
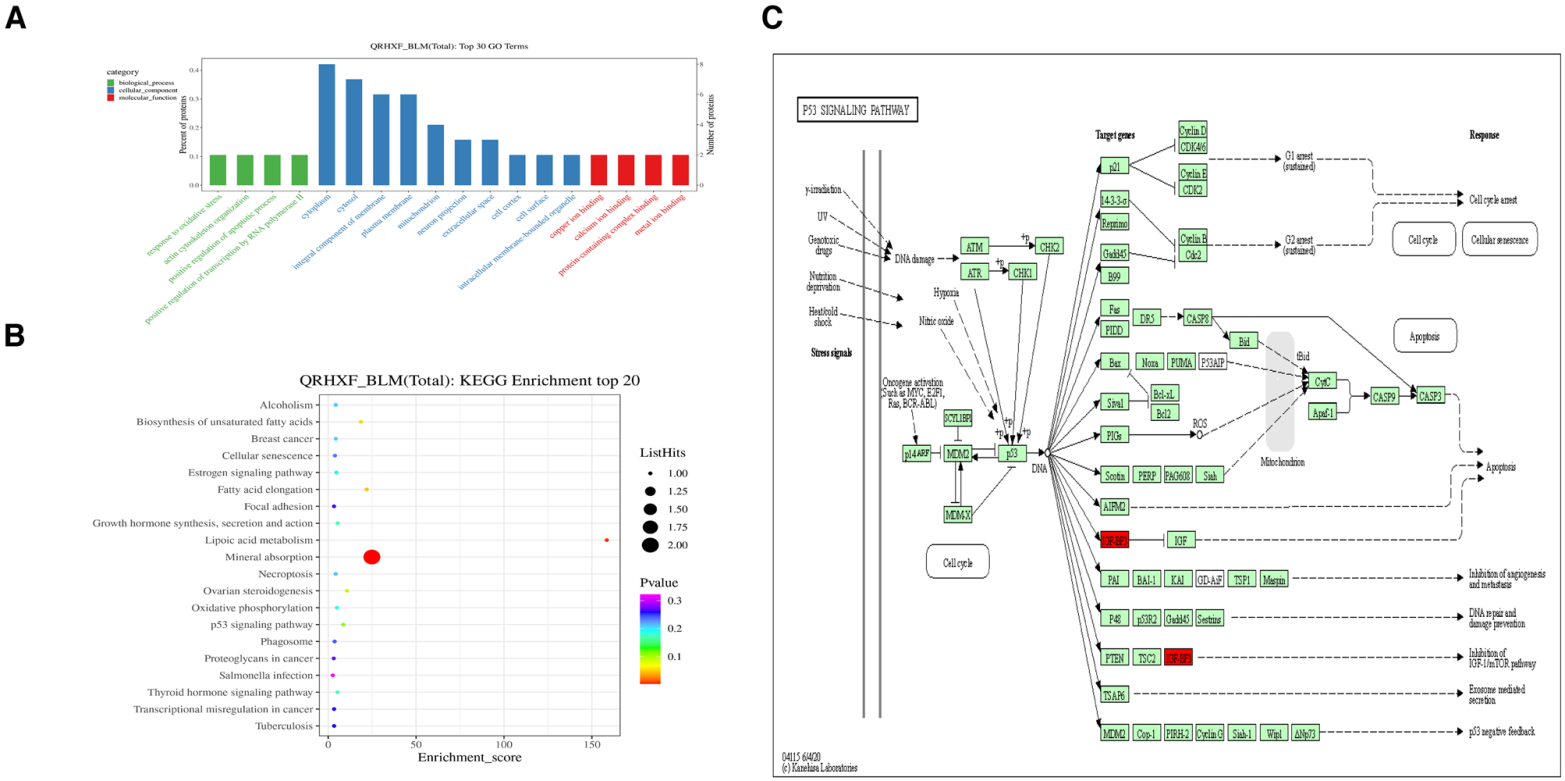
GO and KEGG analysis between the BLM + QRHXF and BLM groups. **A** GO term performance analysis of DEPs between the BLM + QRHXF and BLM groups.** B** The top 20 pathways of KEGG enrichment analysis of DEPs between the BLM + QRHXF and BLM groups.** C** Diagram of the p53 signalling pathway and IGFBP3 (in red colour) between the BLM + QRHXF and BLM groups

For GO analysis, DEPs between the BLM + QRHXF group and BLM group were enriched in the biological process (BP), cellular component (CC), and molecular function (MF) categories (Fig. 7a). The molecular function classification indicated that most of these proteins were involved in protein-containing complex binding. We hypothesized that insulin-like growth factor binding protein 3 (IGFBP3) may be the target protein of QRHXF against pulmonary fibrosis. Pathway enrichment analysis demonstrated enrichment of 20 pathways, including mineral absorption, lipoic acid metabolism, and the p53 signalling pathway, in BLM + QRHXF versus BLM (Fig. 7b). IGFBP3 is enriched and upregulated in the p53 signalling pathway. Therefore, we inferred that QRHXF may attenuate BLM-induced PF through the p53/IGFBP3 signalling pathway (Fig. 7c).

### QRHXF reduces BLM-induced PF partly through the p53/IGFBP3 signalling pathway

qRT-PCR and immunohistochemical staining were conducted to validate the key DEPs and the possible signal transduction pathways identified by TMT quantitative proteomic analysis. Compared with the normal control mice, the expression of p53 and

**Fig. 8:**
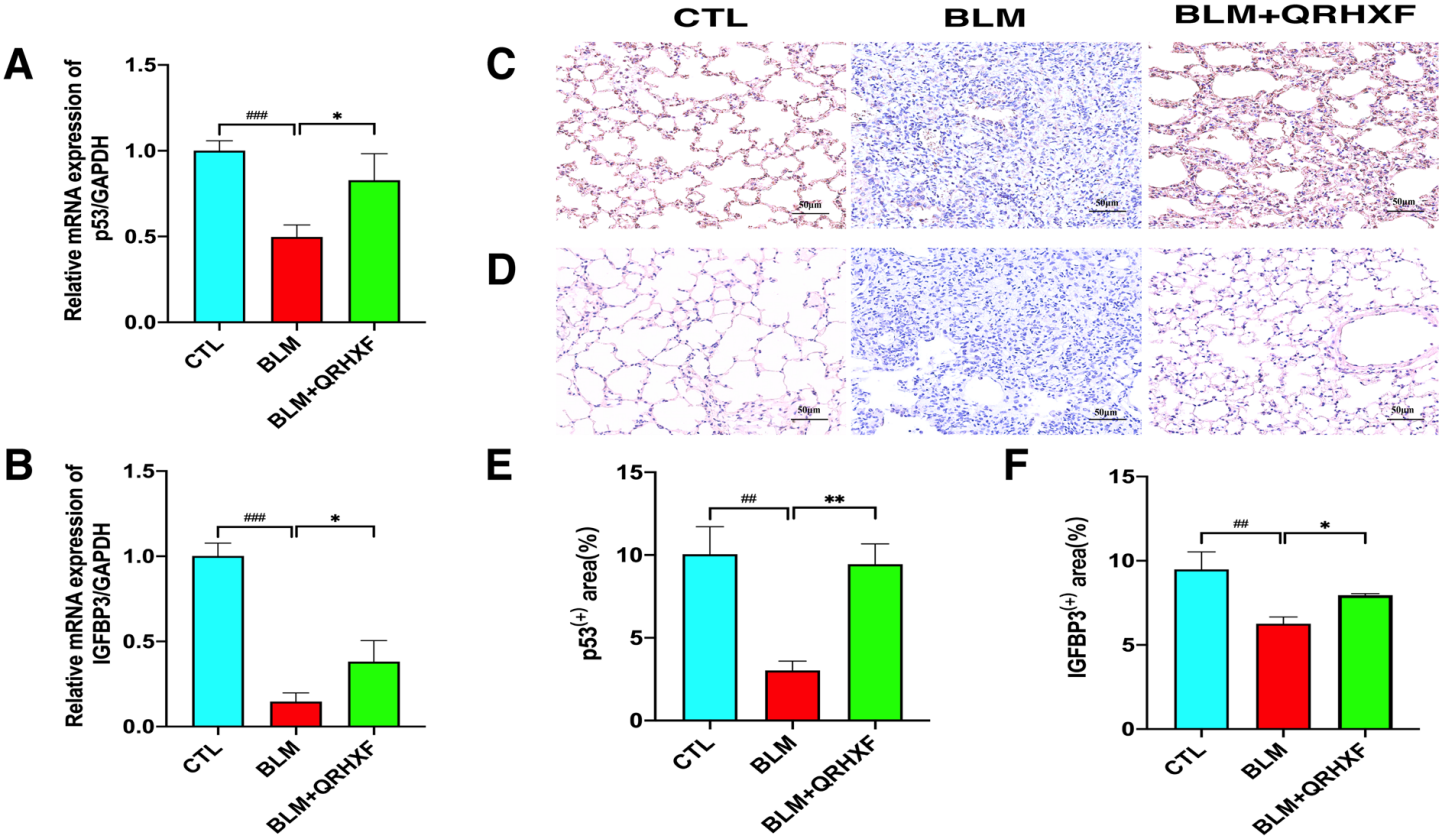
QRHXF reduces BLM-induced PF partly through the p53/IGFBP3 pathway. qRT-PCR validation of the relative mRNA expression of **A** p53 and **B** IGFBP3. Immunohistochemical analysis of protein expression in the lung tissue of each group, including **C** p53 and** D** IGFBP3.** E**, **F** The levels of p53 and IGFBP3 were quantified. n = 4 mice/group, ^##^*p* < 0.001, ^###^*p* < 0.001, compared with the CTL group, *p < 0.05, **p < 0.01, compared with the BLM group. All data are presented as the mean ± SD

IGFBP3 demonstrated an obvious downregulation in BLM-induced PF mice, but treatment with QRHXF significantly upregulated their expression (Fig. 8a–f). Overall, these results implied that the protective effects of QRHXF on PF might be achieved by the regulation of the p53/IGFBP3 pathway.

## Discussion

IPF is a common ILD with progressive and fatal characteristics. Modern medical treatments for IPF are very limited, while TCM has unique advantages in treating this disease. The anti-PF effect of TCM on IPF has been widely studied in recent years. QRHXF is composed of *Scutellaria baicalensis* and *Radix Paeonia Rubra*. In the prescription, *Scutellaria baicalensis*, with bitter flavor and cold property, enters into lung meridian and has the theraputic function of clearing heat, especially heat in upper warmer, purging fire and detoxification. *Radix Paeonia Rubra* is able to reduce heat and cool blood, disperse stasis, and relieve pain. With the complementary of the two drugs, the function of clearing pulmonary heat, promoting blood circulation to dispel blood stasis can be more effective. Our previous research showed that QRHXF and its active components have obvious therapeutic effects on lung diseases such as asthma, COPD and lung cancer [10–12]. It has also been recorded that the active components of QRHXF inhibit PF [14, 15, 22, 23]. In the present study, the BLM-induced PF mouse model was used as the research object, and QRHXF was used for intervention. We found that QRHXF could significantly alleviate weight loss in mice with PF and improve the pulmonary function, lung histopathology and ultrastructural changes of AEC 2 in PF mice (Figs. 2, 3).

High-resolution computed tomography (HRCT) is one of the most important tools for the detection, diagnosis and prognosis of IPF [24]. However, HRCT is a simple structural imaging technique and cannot directly reflect the metabolic situation. Recent advances in technology have brought about the combination of PET with CT, bringing about the fusion of molecular imaging and the accurate structural details of CT [25]. Win T et al. proved that (18)F-FDG PET/CT could predict the mortality of IPF by stratifying the risk factors for IPF patients [26]. Tanguy J et al. also proved that the uptake of (18)F-FDG in the lung was significantly increased in a preclinical model of BLM-induced PF [27]. In the present research, we performed (18)F-FDG PET/CT scans on mice at D27 and found that the model was successful through imaging manifestation. Furthermore, PET/CT was used for the first time to evaluate the effect of traditional Chinese herbal compound prescriptions on PF. Because of the uptake changes of (18)F-FDG in mouse lung tissue and the significant difference in the SUV_mean_ value between the groups (Fig. 2), PET/CT might be practical in assessing the effectiveness of drug treatment.

Some researchers believe that IPF is a chronic inflammatory disease [28, 29]. Various inflammatory cells and cytokines, especially IL-1, IL-6, and TNF-α, have been described to play an important role in the occurrence and progression of PF [30–32]. TGF-β1 is currently the most well-studied profibrotic cytokine and is considered a pivotal factor participating in the development of PF. In addition, studies have also identified that TGF-β1 is a potent inducer of EMT [33, 34]. According to research, EMT is an important pathogenesis of PF, and inhibiting EMT may be an important method to treat PF [35]. Our study demonstrated for the first time that QRHXF has the capacity to alleviate BLM-induced PF in mice. Moreover, we found that QRHXF markedly reduced the expression of inflammatory cytokines, including IL-1β, IL-6, and TNF-α, which increased in mice with PF [36]. Furthermore, with the decreased expression of α-SMA, Col1a1, and vimentin and increased level of E-cadherin in QRHXF-treated mice, we also discovered that the mechanism of inhibition of PF by QRHXF treatment might be related to the attenuation of EMT.

Proteomics was first proposed by Australian scholars Wilkins and Williams in 1994, and its rapid development has ushered in a new era in disease research [37]. Proteomics plays a significant role in the early diagnosis, prognosis, and understanding of the pathogenesis of diseases. In addition, it is also important for pharmaceutical exploitation as a target molecule [38]. Currently, TCM is considered to be nonobjective, lacking in accuracy and reproducibility. Proteomics research can reveal molecular targets and illuminate the potential mechanisms of TCM treatment, thus helping to investigate the scientific connotation of TCM [39, 40]. To elucidate the potential target molecules behind the phenomenon, we analysed the differential protein expression between the CTL, BLM, and BLM + QRHXF groups to explore the anti-PF mechanism of QRHXF by using the LC‒MS/MS-TMT technique. A total of 1919 proteins were quantifiable by proteomics analysis in the three groups, with 1692 DEPs between the BLM and CTL groups, 35 DEPs between the BLM + QRHXF and BLM groups, and 192 DEPs between the BLM + QRHXF and CTL groups. We identified 19 proteins regulated by QRHXF treatment. According to the GO analysis of DEPs, protein-containing complex binding in the molecular function category and extracellular in the cellular component category might be related to the regulatory roles of QRHXF against pulmonary fibrosis. Based on the analysis, IGFBP3 was identified as an important molecule (Figs. 6, 7).

Insulin-like growth factor-binding proteins (IGFBPs) are a family of six highly conserved IGFBPs (IGFBP1 to IGFBP6) that bind to IGF-I and IGF-II with very high affinity, constituting the insulin-like growth factor (IGF) system [41]. Moreover, IGFBP3, the largest and most abundant circulating IGFBP, was initially found to be associated with breast cancer [42]. Researchers have found that IGFBP3 is also associated with some fibrotic diseases, including lung and liver fibrosis. [43, 44]. Melone MA et al. revealed that the IGFBP3 expression level was low in proliferating fibroblasts [45]. A study by Martínez-Castillo M et al. showed that IGFBP3 was downregulated in patients with liver fibrosis [46]. Our experiments revealed that IGFBP3 levels were significantly downregulated in BLM-induced PF mice and could be upregulated by QRHXF treatment (Table 1, Fig. 8).

IGFBP3 is a p53 tumour suppressor-regulated protein [47]. Combined with KEGG pathway analysis, we found that the p53 signalling pathway is also one of the top pathways, and IGFBP3 is also enriched in this pathway. The study by Wu Q et al. suggests that p53 may be an important gene in the occurrence and progression of PF by regulating EMT, senescence, apoptosis, and other cellular processes [48]. Some researchers believe that the expression of p53 is upregulated in IPF [49, 50]. However, Wang m et al. found that the p53 protein expression was significantly downregulated in BLM-induced PF mice and could be upregulated by astaxanthin intervention [51]. We investigated whether QRHXF improves PF via the p53/IGFBP3 pathway in BLM-induced PF mice. The results of immunohistochemistry and qRT-PCR indicated that the expression of p53 and IGFBP3 was downregulated in the BLM model group and upregulated after QRHXF intervention. To summarize, these results demonstrate that the protective effects of QRHXF on PF might be achieved by the regulation of the p53/IGFBP3 pathway (Fig. 8).

However, there are several limitations to this study. First, although one-time intratracheal instillation of BLM in mice is the most classic and commonly used method for modelling PF, it is not without limitations. For example, its fibrosis extends from the periphery to the centre of the lung, and it is also self-healing. Although the most classic BLM-induced mouse PF model was also used in this study, there is still a substantial lack of data sharing in this field. Second, there is an obvious inflammatory stage and a fibrosis stage in the process of BLM-induced PF [52]. Imaging evaluation was performed only in mice at the fibrosis stage, and we do not know whether the antifibrotic effect is achieved by anti-inflammation. To confirm this, a PET/CT examination can be performed in the inflammatory stage to better dynamically assess whether the antifibrotic effect of QRHXF is achieved through anti-inflammatory effects. Third, cell experiments and large-scale clinical randomized controlled trials (RCTs) are needed to corroborate the effectiveness and safety of QRHXF.

## Conclusion

In conclusion, our present study demonstrated that QRHXF treatment could attenuate the BLM-induced PF model in mice. The protective effects of QRHXF on PF might be achieved by the regulation of the p53/IGFBP3 pathway. The present results demonstrate that QRHXF might be a potential therapeutic remedy for PF.

## Supplementary Information

**Additional file 1. Fig. S1**: Total ion chromatography (A) of the Qing-Re-Huo-Xue formula by HPLC-Q/TOF MS.

**Additional file 2: Fig. S2**: Calibration curves, detected ions and test ranges of the 8 compounds in Qing-Re-Huo-Xue formula.

**Additional file 3. Fig. S3**: Heatmap of the levels of DEPs showing hierarchical cluster in CTL, BLM, and BLM + QRHXF groups.

**Additional file 4: Table S1**: Primer sequences.

**Additional file 5: Table S2**: Screening results of the differentially expressed proteins.

## Data Availability

The original contributions presented in this study were included in the article, and further inquiries should be directed to the corresponding authors.

## Abbreviations

AEC2: Alveolar epithelial type II cells
α-SMA: Alpha-smooth muscle actin
BALF: Bronchoalveolar lavage fluid
BLM: Bleomycin
Cchord: Chord compliance
CT: Computed tomography
DEPs: Differentially expressed proteins
ELISA: Enzyme linked immunosorbent assay
EMT: Epithelial-mesenchymal transformation
FC: Fold changes
FDA: Food and Drug Administration
FVC: Forced vital capacity
GO: Gene ontology
HPLC: High performance liquid chromatography
H&E: Hematoxylin and Eosin
IGFBP3: Insulin-like growth factor binding protein 3
IC: Inspiratory capacity
IL-1: Interleukin-1
IL-6: Interleukin-6
ILD: Interstitial lung disease
IPF: Idiopathic pulmonary fibrosis
KEGG: Kyoto Encyclopedia of Genes and Genomes
LC–MS: Liquid chromatography–mass spectrometry
PCR: Polymerase chain reaction
PEF: Peak expiratory flow
PET: Positron Emission Computed Tomography
PF: Pulmonary fibrosis
PFD: Pirfenidone
QRHXF: Qing-Re-Huo-Xue formula
TEM: Transmission electron microscope
TGF-β1: Transforming growth factor-β1
TMT: Tandem mass tags
TNF-α: Tumor necrosis factor-α
