# Supplementary figures and images for: Protective effects of Qing-Re-Huo-Xue formula on bleomycin-induced pulmonary fibrosis through the p53/IGFBP3 pathway
Source: Chin Med. 2023 Mar 30;18:33. doi: 10.1186/s13020-023-00730-y (PMC10061820; doi:10.1186/s13020-023-00730-y)

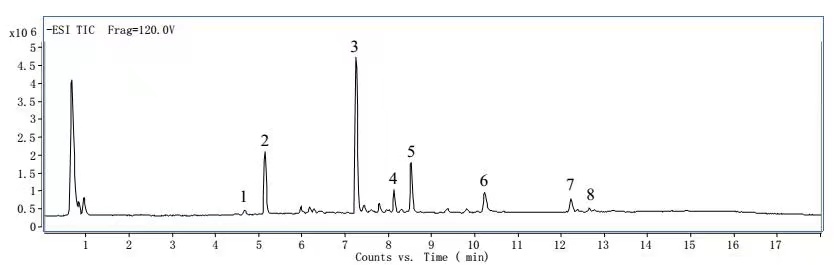

Supplement: Supplementary file 1 — Additional file 1. Fig. S1: Total ion chromatography (A) of the Qing-Re-Huo-Xue formula by HPLC-Q/TOF MS. [file 13020_2023_730_MOESM1_ESM.jpg]

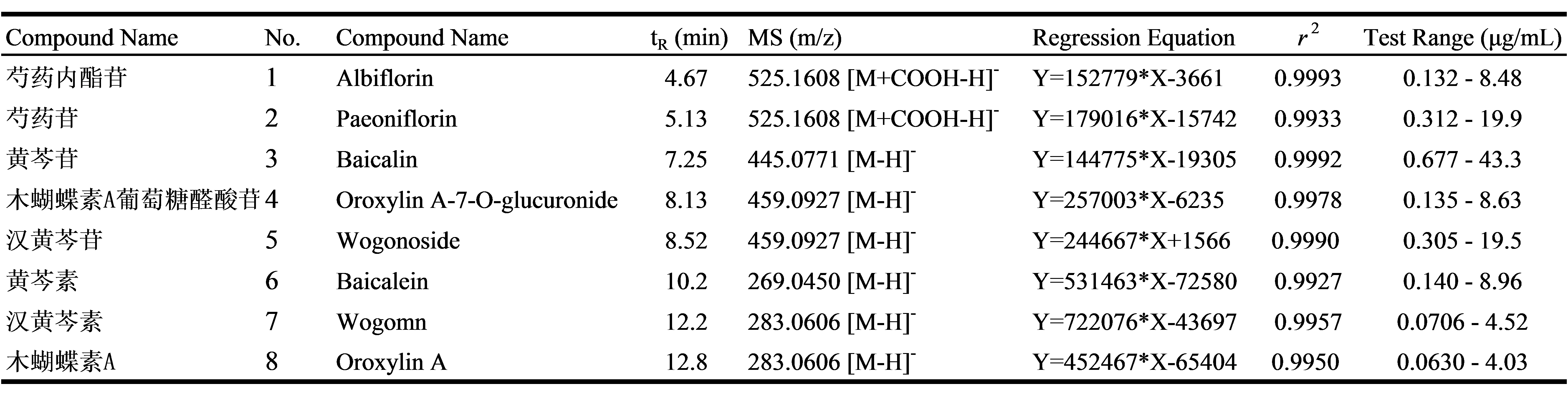

Supplement: Supplementary file 2 — Additional file 2: Fig. S2: Calibration curves, detected ions and test ranges of the 8 compounds in Qing-Re-Huo-Xue formula. [file 13020_2023_730_MOESM2_ESM.png]

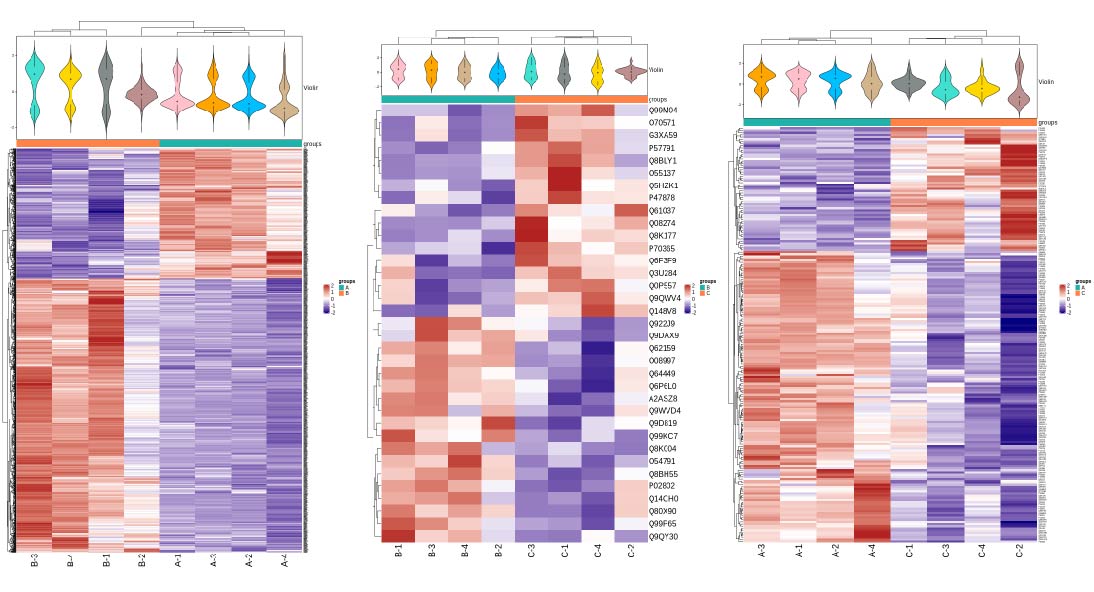

Supplement: Supplementary file 3 — Additional file 3. Fig. S3: Heatmap of the levels of DEPs showing hierarchical cluster in CTL, BLM, and BLM + QRHXF groups. [file 13020_2023_730_MOESM3_ESM.jpg]
