# Supplementary material for: Protective effects of Qing-Re-Huo-Xue formula on bleomycin-induced pulmonary fibrosis through the p53/IGFBP3 pathway
Source: Chin Med. 2023 Mar 30;18:33. doi: 10.1186/s13020-023-00730-y (PMC10061820; doi:10.1186/s13020-023-00730-y)
Supplement: Supplementary file 4 — Additional file 4: Table S1: Primer sequences. [file 13020_2023_730_MOESM4_ESM.docx]

Supplementary Table S1. The sequences of primers

| Name | forward | Reverse |
| --- | --- | --- |
| *Acta2* | CTCTGCCTCTAGCACACA ACT | CCACGAGTAACAAATCAAAGC |
| *Col1a1* | TGACTGGAAGAGCGGAGAGTA | GACGGCTGAGTA GGG AACAC |
| *Vimentin* | CTTCAAGACTCGGTGGACTTC | AGTTGGCAAAGCGGTCATT |
| *CDH1* | TGTCCATGTATTCCTGAAGCC | GCCACACACAGCATAGTCTCA |
| *Trp53* | TCCTCCCCAGCATCT TATC | GAAAAGTCTGCCTGTCTTCC |
| *IGFBP3* | GACACCAAGGGGAAAGACGA | ATGTGGCACGGAGCATCTACT |
| *GAPDH* | AAATGGTGAAGGTCGGTGTG | AGGTCAATGAAGGGGTCGTT |
